# Optimizing manufacturing and composition of a TLR4 nanosuspension: physicochemical stability and vaccine adjuvant activity

**DOI:** 10.1186/1477-3155-11-43

**Published:** 2013-12-21

**Authors:** HW Millie Fung, Traci JT Mikasa, Julie Vergara, Sandra J Sivananthan, Jeffrey A Guderian, Malcolm S Duthie, Thomas S Vedvick, Christopher B Fox

**Affiliations:** 1 IDRI, 1616 Eastlake Ave E, Ste 400, Seattle, WA 98102, USA

## Abstract

**Background:**

Nanosuspensions are an important class of delivery system for vaccine adjuvants and drugs. Previously, we developed a nanosuspension consisting of the synthetic TLR4 ligand glucopyranosyl lipid adjuvant (GLA) and dipalmitoyl phosphatidylcholine (DPPC). This nanosuspension is a clinical vaccine adjuvant known as GLA-AF. We examined the effects of DPPC supplier, buffer composition, and manufacturing process on GLA-AF physicochemical and biological activity characteristics.

**Results:**

DPPC from different suppliers had minimal influence on physicochemical and biological effects. In general, buffered compositions resulted in less particle size stability compared to unbuffered GLA-AF. Microfluidization resulted in rapid particle size reduction after only a few passes, and 20,000 or 30,000 psi processing pressures were more effective at reducing particle size and recovering the active component than 10,000 psi. Sonicated and microfluidized batches maintained good particle size and chemical stability over 6 months, without significantly altering *in vitro* or *in vivo* bioactivity of GLA-AF when combined with a recombinant malaria vaccine antigen.

**Conclusions:**

Microfluidization, compared to water bath sonication, may be an effective manufacturing process to improve the scalability and reproducibility of GLA-AF as it advances further in the clinical development pathway. Various sources of DPPC are suitable to manufacture GLA-AF, but buffered compositions of GLA-AF do not appear to offer stability advantages over the unbuffered composition.

## Introduction

TLR ligands represent an important class of immunomodulators that are utilized to enhance and shape immune responses to recombinant vaccine antigens. In fact, the TLR4 ligand monophosphoryl lipid A (MPL®), a component of the human papilloma virus vaccine Cervarix®, is one of the few adjuvant molecules employed in an FDA-approved human vaccine. The importance of formulation effects on the stability and biological activity of TLR ligands is increasingly being recognized [1,2]. Moreover, differences in vaccine antigen structures may necessitate adjuvant formulation development to optimize antigen-adjuvant compatibility and immunogenicity [3]. We have developed various clinical and preclinical nanoformulations of the synthetic TLR4 ligand glucopyranosyl lipid adjuvant (GLA) [2,4-6]. Since GLA is hydrophobic, appropriate formulation is critical to stably disperse the molecule in aqueous solution. A widely used approach for similarly insoluble molecules is to create a nanoscale aqueous suspension by adding a surfactant, such as a phospholipid, to the insoluble molecule before rehydrating in water. Aqueous nanosuspensions of vaccine adjuvants have shown immunological potency with various antigens, and have progressed to clinical trials [7]. Furthermore, they contain few excipients and are straightforward to manufacture. From a global health perspective, aqueous nanosuspensions present a very appealing option as a vaccine adjuvant product due to low manufacturing cost.

We have developed an aqueous nanosuspension of GLA (GLA-AF) prepared by first co-dissolving GLA and 1,2-dipalmitoyl-*sn*-glycero-3-phosphocholine (DPPC) at a 1:2 molar ratio in chloroform. The organic solvent is then evaporated, leaving a film of the TLR ligand and phospholipid. Finally, water is added to the film and the resulting suspension is created via bath sonication at ~60°C until the formulation is transparent. Previous studies indicate that GLA-AF particle structure is somewhat heterogeneous, consisting of micelles, disks, and vesicles [8,9]. This heterogeneous composition may in part be the result of the sonication manufacturing process. While sonication is convenient for small batch sizes (~10 ml), sonication parameters may not be straightforward to scale up and energy input may not be uniform throughout the sonicated batch. Larger batch sizes or concentrated GLA-AF suspensions may require ≥4 hours of sonication and heating, which is undesirable since prolonged processing and heating of some suspensions may result in component degradation [10]. An alternative formulation processing method is a form of high pressure homogenization called microfluidization. Microfluidization relies on shear forces generated by passing the formulation through narrow orifices at high pressure. This method may reduce processing time compared to sonication; in addition, the microfluidization interaction chamber provides a controlled enforcement of particle size decrease, which may result in a more homogeneous formulation and reduce the potential for component degradation due to over-processing [11]. Furthermore, processing by microfluidization allows for straightforward scale up, and can more easily be tailored for sterile production than sonication [11].

This study aims to optimize GLA-AF by replacing sonication processing with a microfluidization-based approach. We also investigate the effects of different buffer compositions and DPPC from several different suppliers. In order to develop a new composition and/or manufacturing method for GLA-AF, we determined that it must meet or exceed the expected physical and chemical stability, and retain the same biological activity, as GLA-AF prepared in the traditional manner as described above. In order to examine the interactions between the TLR ligand, phospholipid, and aqueous phase, extensive analytical characterization is necessary. We have employed a suite of analytical techniques that are useful for analysis of nanosuspensions, including dynamic light scattering (DLS), HPLC, and differential scanning calorimetry (DSC), which together provide essential data regarding particle size, size polydispersity, component concentration and purity, chemical stability, and lipid phase conformation. Besides evaluating the stability of the formulations produced, we have investigated their innate adjuvant activity on *in vitro* human cells as well as their *in vivo* biological activity as vaccine adjuvants with a model recombinant antigen in the murine model.

## Materials and methods

### Adjuvant formulation materials and manufacture

Synthetic monophosphoryl lipid A (glucopyranosyl lipid adjuvant or GLA) was purchased from Avanti Polar Lipids Inc. (Alabaster, AL). 1,2-dipalmitoyl-*sn*-glycero-3-phosphatidylcholine (DPPC) was purchased from four different suppliers: Avanti Polar Lipids Inc. (Alabaster, AL), Corden Pharma (Liestal, Switzerland), Lipoid LLC (Newark, NJ), and NOF Corporation (Kawasaki, Japan). Monobasic and dibasic ammonium phosphate, monobasic potassium phosphate, dibasic sodium phosphate, and monohydrate citric acid were purchased from J.T. Baker (San Francisco, CA). Phosphate buffered saline 1× (PBS) at pH 7.2 was purchased from Invitrogen (Grand Island, NY). Sodium chloride and HEPES were purchased from Sigma-Aldrich (St. Louis, MO).

GLA-AF at 0.25 or 1 mg/ml GLA was manufactured by first combining GLA and DPPC at a 1:2 molar ratio (GLA:DPPC) in excess chloroform in 10-ml vials, which were then evaporated using a Genevac EZ-2 Plus Evaporator (Stone Ridge, New York) or a Buchi Rotavapor R-114 (Flawil, Switzerland). Vials containing the dried DPPC and GLA were rehydrated in 10 ml ultrapure water per vial, then sonicated in a VWR 75D (West Chester, PA) or Crest Powersonic CP230D (Trenton, NJ) sonicating water bath at ~60°C for ~1-2 hours or until the formulation was translucent with no visible particles. All buffered GLA-AF formulations were manufactured at 1 mg/ml GLA. Manufacture procedure is the same as above, except the dried DPPC and GLA were rehydrated in 10 ml of the following buffers instead of ultrapure water: ammonium phosphate (5, 12.5, 20, 25 mM, pH ~5.5); citric acid (10 mM, pH 6.1); phosphate-buffered saline (composed of 1.5 mM monobasic potassium phosphate, 155 mM sodium chloride, and 2.7 mM dibasic sodium phosphate, at pH 7.2; or 41 mM monobasic potassium phosphate, 103 mM sodium chloride, and 9 mM dibasic sodium phosphate, at pH 6.1); HEPES (10 mM, pH 7); and HEPES-buffered saline (10 mM HEPES, 154 mM NaCl, pH 7). Buffer compositions (and pH values) were selected based on existing clinical adjuvant formulations or in related literature of interest. Thus, ammonium phosphate is the buffer employed in IDRI’s emulsion adjuvant (SE or GLA-SE) currently in clinical trials [6]. The 10 mM citrate buffer is the buffer in Novartis’ emulsion adjuvant, MF59® [12]. The PBS formulation with pH 6.1 is the buffer used in GlaxoSmithKline’s liposome adjuvant, AS01 [13]. The 10 mM HEPES buffers were motivated based on favorable performance (compared to PBS) in the manufacture of DPPC dispersions reported previously [14]. The ammonium phosphate buffer was prepared at four different strengths to evaluate the effect of buffer strength. The sonicated formulations were filtered using a Pall Life Sciences, Acrodisc® syringe filter, 0.2-μm Supor® membrane (Port Washington, NY).

Aqueous formulations made by microfluidization were prepared in a similar way as described above up to the point of chloroform evaporation on the Genevac EZ-2 Plus Evaporator (Stone Ridge, New York). The vial containing the lipid film was rehydrated with 10 ml ultrapure water then briefly sonicated (~5 minutes) in a VWR 75D (West Chester, PA) or Crest Powersonic CP230D (Trenton, NJ) sonicating water bath at 60°C until the dried GLA-DPPC mixture was removed from the sides of the glass vial. The pre-processed formulation was diluted with ultrapure water to reach final theoretical concentrations of 0.25 mg/ml, 0.1 or 0.025 mg/ml GLA. The Microfluidics M110P (Newton, MA), equipped with a diamond F12Y interaction chamber followed in series by a ceramic H30Z auxiliary processing module, was employed for processing the formulations at three different pressures: 10,000, 20,000, and 30,000 psi for up to 20 discrete passes with recirculating chilled water to prevent excessive temperature increase during processing. To determine the optimum processing conditions for manufacturing GLA-AF, the 0.1 mg/ml GLA-AF was processed at 3 different pressures and 50-μl aliquots were removed after each pass up to 20 passes for particle size characterization by dynamic light scattering as described below. The remaining formulation after the 20th pass for each pressure was placed on a standard stability measurement schedule at the time of manufacture and 1 week, 2 week, 1 month, 3 months, and 6 months thereafter to monitor particle size and GLA concentration. Buffered GLA-AF (12.5 mM ammonium phosphate) was also prepared by microfluidization. Buffered GLA-AFs were prepared in the same way as the unbuffered GLA-AF up to the point of rehydration. The ammonium phosphate GLA-AF was manufactured at 0.1 mg/ml GLA and processed at 20,000 and 30,000 psi up to 10 passes each on the microfluidizer with aliquots removed at the 2^nd^ and 10^th^ passes for stability assays. Selected microfluidized GLA-AF batches were filtered through a 0.2-μm Supor® membrane prior to *in vivo* biological activity evaluation.

### Dynamic light scattering particle size analysis

Particle size for each formulation was determined using the Malvern Instruments (Worcestershire, UK) Zetasizer Nano-S or –ZS via dynamic light scattering (DLS). 50 μl of GLA-AF were combined with 450 μl ultrapure water in a 1.5-ml polystyrene disposable cuvette. DLS measurements were made three times on each cuvette.

### HPLC-CAD analysis

To prepare GLA-AF samples, the formulation was diluted 1:20 or 1:5 (depending on target GLA concentration) into mobile phase A (75:15:10 [v:v:v] methanol:chloroform:water with 20 mM ammonium acetate and 1% acetic acid). For each formulation, three separate vials were prepared. All samples were injected at 50-μl volume onto a Waters Co. (Milford, MA) XBridge BEH Shield RP18 column attached to an Agilent Model 1100 HPLC (Santa Clara, CA). A gradient consisting of mobile phases A and B (1:1 [v:v] methanol:chloroform with 20 mM ammonium acetate and 1% acetic acid) was employed over 25 minutes. Detection was performed by an ESA Biosciences (Chelmosford, MA) Corona Charged Aerosol Detector (CAD). Quantitation was performed using a GLA standard injected at different volumes in mobile phase B to create a standard curve. Due to assay variation, a +/− 20% of target value is employed as the specification for detecting meaningful change in GLA concentration.

### Differential scanning calorimetry

Thermal phase transition profiles for sonicated and microfluidized 0.1 mg/ml GLA-AF batches were determined using the MicroCal VP-DSC (Northampton, MA). Ultrapure pure water was used as reference for all GLA-AF samples. All references and samples were degassed using the MicroCal ThermoVac (Northampton, MA) at 24°C for 5 minutes before loading into the reference and sample cells. For each lot analyzed, scans were performed in the Identical Scan Mode from 5°C to 60°C at a scan rate of 60°/hr.

### In vitro whole blood stimulation

Formulations were prepared in 1.5-ml capacity eppi-tubes by diluting with phosphate buffered saline (PBS) pH 7.2 purchased from Life Technologies (Grand Island, NY), thereby standardizing the GLA concentration for each formulation at 6 μg in a total volume of 1 ml. 150 μl of each formulation were plated in duplicate into 96-well round-bottom tissue culture plates, followed by seven 1:3 serial dilutions in PBS, resulting in final well volumes of 100 μl and spanning a GLA concentration range between 1 – 3000 ng/ml. Next, 100 μl of heparinized whole blood (obtained with informed consent from three healthy volunteers) were added to each well, then incubated at 37°C and 5% CO_2_ for 24 hrs. After incubation, the plates were microcentrifuged for ten minutes at 1600 RPM and two 50-μl extractions of the plasma supernatant from each well were carefully obtained and assayed for cytokine content per manufacturer’s instructions, using ELISA kits for MIP-1β and IL-12p40 from R&D Systems (Minneapolis, MN). Standards were prepared in triplicate (plated separately from the formulation-stimulated supernatants) according to the respective ELISA protocols, spanning a range of 0.008 – 1 ng/ml for MIP-1 β and 0.031 – 4 ng/ml for IL-12p40.

### In vitro mono mac 6 cell line stimulation

Cell culture, maintenance and *in vitro* stimulation set-up of the human acute monocytic leukemia cell line from Leibniz-Institute DSMZ (Braunschweig, Germany) were performed as instructed by the supplier’s protocol up to a week in advance of planned stimulation. Formulations were prepared in 1.5-ml capacity eppi-tubes by diluting with Mono Mac 6 cell media, standardizing the GLA concentration for each formulation at 6 μg in a total volume of 833 μl. 180 μl of each formulation prep were plated in duplicate into 96-well round-bottom tissue culture plates, followed by seven 1:3 serial dilutions with Mono Mac 6 cell media, giving final well volumes of 120 μl and spanning a GLA concentration range between 1 – 3000 ng/ml. Next, 100 μl from each well were transferred to a separate well pre-plated with 100 μl (1–2 × 10^5^ cells) of Mono Mac 6 cells and incubated at 37°C and 5% CO_2_ for 24 hrs. After incubation, the plates were microcentrifuged for ten minutes at 1600 RPM and 75 μl of the supernatant were extracted from each well and assayed for cytokine content using the ELISA kit from R&D Systems (Minneapolis, MN) for MIP-1β. Standards template applied to this assay was obtained from the MIP-1β ELISA run described above for the whole blood stimulation assay.

### Mouse immunizations and immune response analysis

*Plasmodium berghei* circumsporozoite protein (PbCSP) was expressed and purified from *E. coli* using the codon-harmonized construct kindly provided by Dr. Evelina Angov from the Walter Reed Army Institute of Research. All animal protocols were approved by the IDRI institutional animal care and use committee. Female C57BL/6 mice were purchased from Charles River Laboratories (Wilmington, MA) and maintained in specific pathogen-free conditions. Mice, 6–8 weeks of age and 4–5 mice per group, were immunized a total of three times at two-week intervals by injection into the quadricep. For immunization, recombinant protein was formulated with adjuvant to provide a total of 2 μg protein/injection with 1 μg GLA in a total volume of 0.1 ml.

Blood was collected from the retro-orbital sinus two weeks after the second immunization and processed using Microvette 200 Z-Gel (SARSTEDT AG & Co., Numbrecht, Germany) according to the manufacturer's recommendation. Sera were subsequently stored at 4°C until antigen-specific antibody responses were determined by ELISA. Corning 384-well High Binding Plates (Corning, Rochester, NY) were coated overnight with 1 μg/ml PbCSP antigen in Ebioscience 0.1 M PBS coating buffer. Plates were washed using PBS/0.05% Tween 20 and blocked greater than 2 hours at room temperature with 1% BSA-PBS 0.05% Tween. Following washes in PBS/Tween, mouse serum was added and serially diluted starting with first well (1:100) 1 to 4 in plate using Saigen Multipette® 96 channel Robotic platform. After primary incubation and further washes, either anti-mouse IgG-HRP, anti-mouse-IgG2c-HRP or anti-mouse IgG1-HRP were added (all Southern Biotech, Birmingham, AL). After 1 hour incubation at room temperature and washing, Sure Blue™ TMB (Kirkegaard and Perry Laboratories, Gaithersburg, MD) microwell Peroxidase was added to each well to reveal reactions, which were then stopped by the addition of 1 N H2SO4. Plates were analyzed at 450-570 nm (Synergy HT, Bio-Tek Instruments Inc, Winooski, VT). Endpoint titer was determined by Graph Prism nonlinear regression (curve fit) sigmoidal dose response (variable slope) to interpolate unknowns from the last optical density (OD) value greater than a threshold determined by sera from unimmunized mice.

One month after the final immunization, spleens were removed and single cell suspensions prepared. Mononuclear cells were enumerated using a ViaCount assay with a Guava Easy cyte HT (Guava Technologies, Hayward, CA), resupended in RPMI-1640 supplemented with 10% heat-inactivated FBS and 100 Units penicillin/streptomycin (Gemini Bio-Products). To measure cytokine production, mononuclear cells were incubated at 2 × 10^5^ cells per well in duplicate in a 96-U Bottom well plate (Corning Incorporated, Corning, NY) in the presence of 10 μg/ml protein for 4 days, after which supernatants were collected and IFNγ/IL-5 content determined by ELISA according to the manufacturer’s instructions (eBioscience, San Diego, CA).

To determine the number of cells producing each cytokine, multiScreen 96-well filter plates 0.45 μm Hydophobic High Protein Binding Immobion-P Membrane (Millipore) were coated with rat anti-mouse IL-5 or rat anti-mouse IFNγ capture antibody (both eBioscience) according to manufacturer’s recommendation and incubated overnight at 4˚C. Plates were washed with PBS, blocked with RPMI 1640 containing 10% FBS 100 units penicillin/streptomycin for at least 1 h 37˚C, and washed again. Spleen cells were then added at 2 × 10^5^ cells/well (100 ul) and incubated with media, PbCSP (10 μg/ml) or Concavalin A (1.5ug/ml) Sigma (for 48 h at 37°C). The plates were then washed with PBS–0.1% Tween 20 and incubated with a biotin-conjugated rat anti-mouse IL-5 or IFNγ secondary antibody (eBioscience) diluted in 5× Assay Buffer (eBioscience). The filters were developed using the Vectastain AEC substrate kits according to the manufacturer’s protocol (Vector Laboratories, Burlingame, CA). Reactions were stopped by washing with deionized water, then plates were dried in the dark and spots were enumerated using an automated ELISPOT reader (CTL Immunospot; Cellular Technology Ltd., Shaker Heights, OH) and analyzed with ImmunoSpot software (Cellular Technology Ltd).

## Results and discussion

In general, GLA-AF preparations were examined for the following indicators of formulation stability: visual appearance, particle size, and GLA concentration. Particle size, based on scattering intensity (Z-ave) and polydispersity index (PdI), was measured via dynamic light scattering. GLA concentration was measured via HPLC with charged aerosol detection (CAD). Visual appearance and particle size were examined on the date of manufacture (DM), and at the following timepoints after the DM: 1 week, 2 weeks, 1 month, 3 months, and 6 months. GLA concentration was measured on the DM and at 6 months. We considered GLA-AF to be unstable if at least one of the following conditions occurred for each batch of manufactured formulation: visual appearance that was not indicative of homogeneous, translucent solution (i.e. presence of large visible particulates); particle size change of more than 50% of the particle size on the DM; GLA concentration change of more than 20% of the GLA concentration on the DM. Upon failing a stability test, the formulation was removed from testing and not measured at subsequent timepoints.

### Influence of DPPC from different manufacturers on physicochemical stability

**Figure 1:**
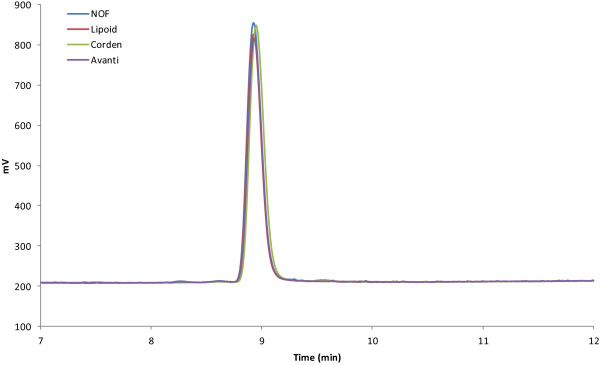
HPLC-CAD profiles of DPPC from four different suppliers indicate comparable raw material purity.

Two separate batches of GLA-AF employing DPPC from each of four different DPPC suppliers were prepared (Avanti Polar Lipids Inc., Corden Pharma, Lipoid LLC, and NOF Corporation). HPLC-CAD traces show only a single peak for the raw material from all four suppliers, indicating that DPPC purity was comparable among the different manufacturers (Figure 1). In addition, the physicochemical stability indicators of all batches of GLA-AF were also comparable. All eight batches were stable according to the criteria described above regarding visual appearance, particle size, and GLA concentration over 6 months (Table 1). Since GLA-AF in this case was processed via bath sonication, particle size was not tightly reproducible between different lots, as expected due to the inherent difficulty with bath sonication. Thus, even when processed in the exactly same manner, different lots containing the same excipients could result in slightly different particle size, but all batches remained in the range of 81–106 nm. There were also slight variations in initial GLA concentrations measured from the eight lots, but those variations show no correlation to DPPC manufacturers.

**Table 1:** Effect of DPPC from different suppliers on GLA-AF physicochemical stability

**DPPC supplier**		**Particle size (Z-ave, nm)**		**Pdl**		**Measured GLA concentration (μg/ml) [250 ± 50 μg/ml target]**	
		**DM**	**6 mo**	**DM**	**6 mo**	**DM**	**6 mo**
NOF	Lot 1	85 ± 2	87 ± 3	0.27 ± 0.01	0.27 ± 0.01	222 ± 6	200 ± 3
	Lot 2	78 ± 1	81 ± 2	0.31 ± 0.03	0.33 ± 0.04	206 ± 2	166 ± 2
Corden	Lot 1	88 ± 3	89 ± 2	0.28 ± 0.03	0.26 ± 0.01	209 ± 10	179 ± 5
	Lot 2	102 ± 1	106 ± 2	0.24 ± 0.01	0.25 ± 0.01	206 ± 3	171 ± 11
Lipoid	Lot 1	101 ± 2	100 ± 2	0.27 ± 0.01	0.25 ± 0.01	230 ± 3	210 ± 3
	Lot 2	86 ± 1	88 ± 2	0.27 ± 0.01	0.27 ± 0.03	212 ± 4	197 ± 2
Avanti	Lot 1	87 ± 1	85 ± 1	0.22 ± 0.01	0.21 ± 0.01	261 ± 4	231 ± 2
	Lot 2	93 ± 1	93 ± 1	0.26 ± 0.01	0.25 ± 0.01	223 ± 2	234 ± 2

### Influence of buffer composition on physicochemical stability

**Figure 2:**
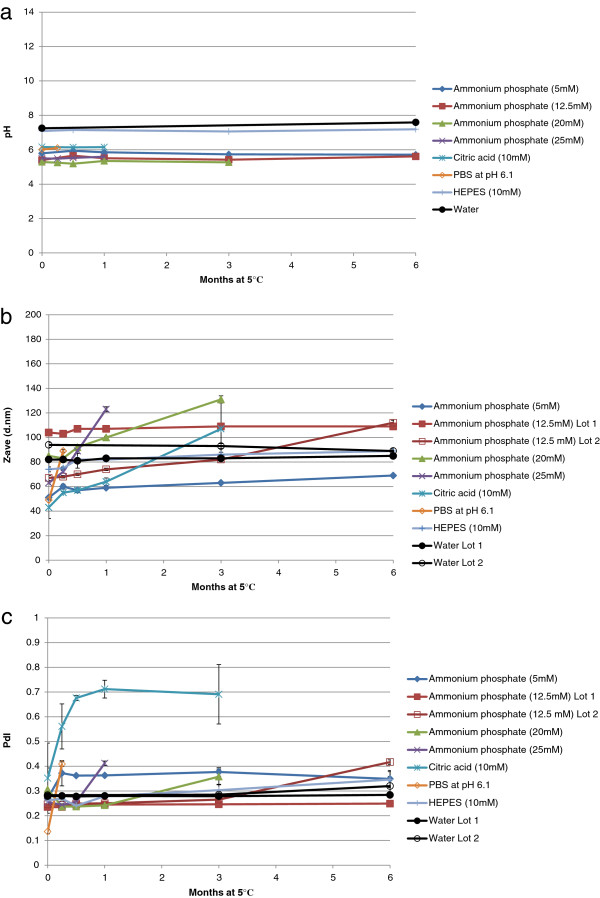
**Characterization of pH and size stability of GLA-AF with various buffer compositions. (a)** pH of buffered GLA-AF’s at the following time points: DM, 1 week, 2 weeks, 1 month, 3 months, 6 months. **(b)** Particle size of buffered GLA-AF compositions measured at DM, 1 week, 2 weeks, 1 month, 3 months, and 6 months. **(c)** Polydispersity index (PdI) of buffered GLA-AF compositions measured at DM, 1 week, 2 weeks, 1 month, 3 months, and 6 months. GLA-AF made with ammonium phosphate at 20 or 25 mM, citric acid, or PBS failed to maintain particle size within 50% of the DM value before reaching the 6-month timepoint, thus pH, size, and PdI data for those lots were measured until (and including) the time point at which their size failed this specification.

Since GLA-AF is formulated by rehydrating the GLA and DPPC mixture in water, the formulation is not protected from fluctuations in pH, which could result in component degradation. In an attempt to maintain pH control, the following buffers were employed as the aqueous phase: ammonium phosphate (5, 12.5, 20, 25 mM, pH ~5.5); citric acid (10 mM, pH 6.1); phosphate-buffered saline (composed of 1.5 mM monobasic potassium phosphate, 155 mM sodium chloride, and 2.7 mM dibasic sodium phosphate, at pH 7.2; or 41 mM monobasic potassium phosphate, 103 mM sodium chloride, and 9 mM dibasic sodium phosphate, at pH 6.1); HEPES (10 mM, pH 7); HEPES-buffered saline (10 mM HEPES, 154 mM NaCl, pH 7). These various buffers were tested to determine the most favorable buffer composition, pH condition, and salt concentration for GLA-AF formation and stability. The formulation pH was monitored to evaluate whether the buffer provided pH control. Along with the standard physicochemical characteristics mentioned above, pH was measured on the DM, at 1 week, 2 weeks, 1 month, 3 months, and 6 months. The pH values of all buffers that were compatible with GLA-AF formation showed no more than a 0.21 difference for 6 months, or up until the formulation became unstable by particle size (Figure 2a). The unbuffered GLA-AF, with ultrapure water as its aqueous phase, showed only a slightly higher pH shift of 0.34 after 6 months, although it is unclear if this magnitude is significant since temperature was not tightly controlled for the pH measurements. Large visible particles still existed in preparations containing GLA and DPPC in PBS at pH 7.2 or HEPES-buffered saline at pH 7 even after 3 hours of sonication, so these formulations were not evaluated further regarding physicochemical characteristics or stability.

There were interesting differences regarding the influence of buffer composition on GLA-AF particle size stability (Figure 2b). GLA-AF made with ammonium phosphate at 20 or 25 mM, citrate, or PBS at pH 6.1 failed before 6 months due to not meeting the particle size criteria of <50% change from the DM value. Likewise, these formulations showed increased PdI values correlating with their increased particle size (Figure 2c). Some buffered and unbuffered formulations were stable with regards to particle size (<50% change) as well as visual appearance, although the particle size of the GLA-AF containing 5 mM ammonium phosphate buffer and the GLA-AF containing 10 mM HEPES indicated gradual increase in size over 6 months. Moreover, some variability between batches of the same composition should be taken into account, since the first batch of GLA-AF buffered at 12.5 mM ammonium phosphate showed little change in size or PdI over 6 months, but a second batch with the same composition demonstrated gradual particle size growth, eventually achieving >50% of initial size by the 6-month timepoint. Chemical stability of GLA changed less than 20% in all formulations that demonstrated stable particle size over 6 months (Table 2). However, the DM GLA concentration for the formulation containing ammonium phosphate buffer at 5 mM was not within 20% of the initial targeted GLA concentration of 1000 μg/ml (possibly indicating some difficulty in efficient dispersion of all of the GLA during sonication).

**Table 2:** GLA concentration of buffered GLA-AF compositions

**Aqueous phase compositions and concentrations (mM)**	**Measured GLA concentration (μg/ml) [1000 ± 200 μg/ml target)**	
	**DM**	**6 mo**
Ammonium phosphate (5)	737 ± 34	715 ± 34
Ammonium phosphate (12.5) Lot 1	861 ± 21	791 ± 21
Ammonium phosphate (12.5) Lot 2	860 ± 24	N/A
Ammonium phosphate (20)	920 ± 13	N/A
Ammonium phosphate (25)	982 ± 15	N/A
Citric acid (10)	886 ± 14	N/A
Phosphate buffered saline (50), pH 6.1	960 ± 18	N/A
HEPES (10)	945 ± 25	912 ± 28
Water Lot 1	864 ± 28	992 ± 8
Water Lot 2	930 ± 14	773 ± 6

### Influence of manufacturing process on physicochemical stability

**Figure 3:**
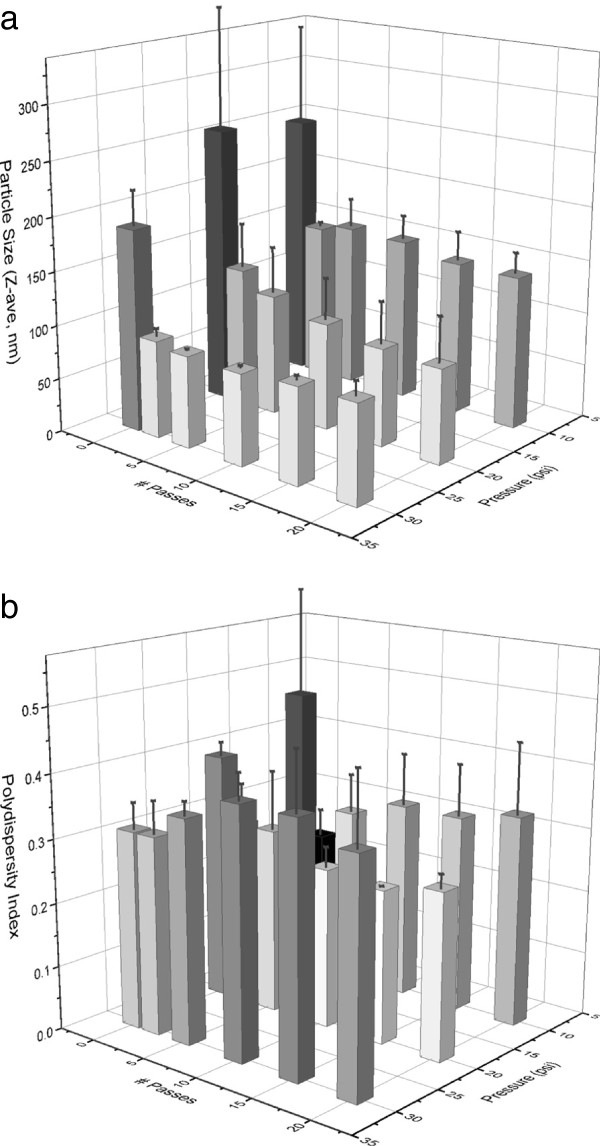
**Microfluidization processing effects on size characteristics of GLA-AF. (a)** Effect of microfluidizing pressure and number of discrete passes on unbuffered GLA-AF particle size (0.1 mg/ml GLA target concentration). **(b)** Effect of microfluidizing pressure and number of discrete passes on unbuffered GLA-AF size polydispersity index. Two batches were manufactured at each pressure and monitored for size and PdI at 0, 2, 5, 10, 15, and 20 passes with the exception of one of the 10,000 psi batches where the 2^nd^ pass measurement was missed so a 3^rd^ batch was manufactured at 10,000 psi and measured at 0, 2, and 5 passes only.

To evaluate the effects of different microfluidization parameters on the physicochemical characteristics of GLA-AF, we focused on the most stable compositions from the buffer evaluation experiment described above, including unbuffered GLA-AF or GLA-AF containing 12.5 mM ammonium phosphate. Various processing pressure and cycling time (i.e. number of passes) conditions were employed, and the resulting effects on particle size and GLA concentration over 6 months monitored for unbufferd GLA-AF (Figure 3, Table 3) and buffered GLA-AF (Table 4). Due to cost and material use concerns associated with the microfluidizing process requiring large minimum batch volumes (~80 ml), GLA-AF was generally manufactured at more dilute concentrations compared to the sonicated batches above. Apparently very few passes are necessary to form small particle sizes (~100 nm) at 20,000 psi or 30,000 psi, whereas higher number of passes did not reproducibly reduce size further and could even result in larger size. Likewise, polydispersity index often increased with higher numbers of passes. Both unbuffered and ammonium phosphate-buffered compositions appeared amenable to microfluidization, although there was some growth in particle size for the buffered GLA-AF. The 10,000 psi batch of unbuffered GLA-AF where GLA was quantified resulted in only ~50% recovery of GLA after 20 passes (the GLA recovery was ~55% after 5 passes, so most of the GLA was lost early in the process). All of the batches manufactured at 20,000 psi or 30,000 psi resulted in successful recovery of the GLA (within 20% of target value, Tables 3 and 4), with the exception of one unbuffered 20,000 psi batch. Furthermore, we attempted manufacture of two batches at the higher target concentration of 0.25 mg/ml GLA processed at 30,000 psi which demonstrated successful GLA recovery but some variation in size between batches (Table 3). We note that lower concentration GLA-AF batches were also successfully manufactured for use in the *in vivo* bioactivity evaluation described below. Thermal phase transition profiles between sonicated and microfluidized GLA-AF batches are broad overall, but sonicated batches appear to have a slightly higher phase transition peak than microfluidized batches, indicating that the phase structure of the GLA-DPPC complexes may be somewhat altered by the different processing conditions (Additional file 1: Figure S1).

**Table 3:** Effect of microfluidization processing on unbuffered GLA-AF recovery and physicochemical stability

**Processing pressure (psi)**	**Target GLA concentration (μg/ml)**	**# Passes**	**Particle size (Z-ave, nm)**		**Pdl**		**Measured GLA concentration**	
			**DM**	**T = 6 mo**	**DM**	**T = 6 mo**	**T = DM or 3 mo**	**T = 6 mo**
10, 000	100	20	128 ± 3	-	0.25 ± 0.03	-	-	-
		20	156 ± 4	-	0.41 ± 0.03	-	50 ± 0	-
20, 000	100	20	58 ± 2	56 ± 1	0.24 ± 0.01	0.23 ± 0.02	90 ± 2	88 ± 4
		20	118 ± 1	-	0.27 ± 0.00	-	72 ± 4	-
30, 000	100	20	81 ± 2	92 ± 1	0.44 ± 0.02	0.47 ± 0.04	85 ± 2	87 ± 3
		20	100 ± 11	92 ± 2	0.28 ± 0.06	0.40 ± 0.02	104 ± 1	84 ± 3
	250	15	133 ± 3	-	0.29 ± 0.01	-	231 ± 8	-
	250	15	58 ± 1	-	0.25 ± 0.02	-	232 ± 6	-

**Table 4:** Effect of microfluidization processing on GLA-AF recovery and physicochemical stability when buffered with 12.5 mM ammonium phosphate

**Processing pressure (psi)**	**# Passes**	**Particle size (Z-ave, nm)**		**Pdl**		**Measured GLA concentration [100 ± 20 μg/ml target]**	
		**DM**	**T = 6 mo**	**DM**	**T = 6 mo**	**DM**	**T = 6 mo**
20, 000	2	99 ± 3	108 ± 2	0.21 ± 0.01	0.32 ± 0.01	89 ± 1	96 ± 5
	5	99 ± 4	-	0.25 ± 0.02	-	-	-
	10	96 ± 1	124 ± 5	0.26 ± 0.01	0.53 ± 0.02	113 ± 4	94 ± 2
30, 000	2	99 ± 3	93 ± 1	0.21 ± 0.01	0.37 ± 0.02	89 ± 2	96 ± 5
	5	67 ± 1	-	0.22 ± 0.01	-	-	-
	10	68 ± 1	84 ± 2	0.27 ± 0.02	0.37 ± 0.02	105 ± 1	88 ± 3

### In vitro biological activity

**Figure 4:**
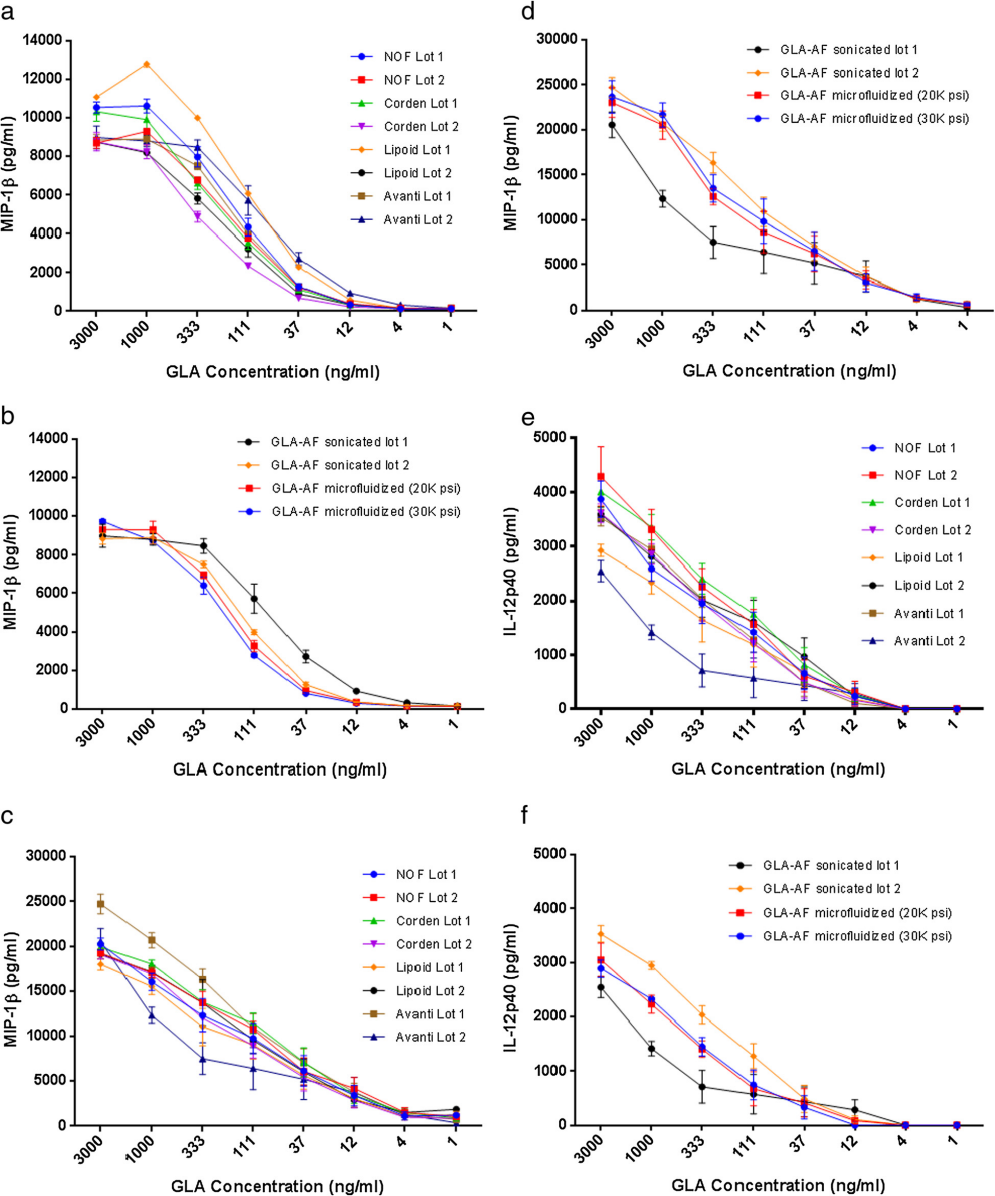
***In vitro *cytokine production activity elicited by GLA-AF. (a)** MIP-1β production by Mono Mac 6 cells stimulated with unbuffered GLA-AF manufactured with DPPC from different suppliers. **(b)** MIP-1β production by Mono Mac 6 cells stimulated with unbuffered GLA-AF manufactured by different processes. **(c)** MIP-1β production by human whole blood stimulated with unbuffered GLA-AF manufactured with DPPC from different suppliers. **(d)** MIP-1β production by human whole blood stimulated with unbuffered GLA-AF manufactured by different processes. **(e)** IL-12p40 production by human whole blood stimulated with unbuffered GLA-AF manufactured with DPPC from different suppliers. **(f)** IL-12p40 production by human whole blood stimulated with unbuffered GLA-AF manufactured by different processes. For all plots, error bars represent standard error of the mean, based on six values (sera from three volunteers assayed in duplicate) and note that one of the GLA-AF batches employed in these studies is not the same one represented in Table 1.

Macrophage inflammatory protein-1β (MIP-1β; also known as CCL4) is a chemoattractant for various immune cells. The production of MIP-1β by the human macrophage cell line, Mono Mac 6, is being developed in our laboratory as a quality control assay for TLR4 agonists due to linearity and reproducibility [9]. Consistent with the similar physicochemical characteristics measured for each distinct formulation, no differences were observed in MIP-1β production by Mono Mac 6 cells stimulated either by batches of GLA-AF made with DPPC from four different suppliers (Figure 4a) or by different processes (Figure 4b). Stimulation of unfractionated human blood yielded similar results in terms of MIP-1β production (Figure 4c and 4d). Production of IL-12, a cytokine critical for development of antigen-specific inflammatory responses was also comparable across the different GLA-AF formulations (Figures 4e and 4f). There was an indication of greater batch variation in the sonicated GLA-AF than the microfluidized GLA-AF. Refined evaluation of additional batches is required to assess this possibility, which could indicate that microfluidization produces a more uniform, reproducible formulation than sonication.

### In vivo biological activity

**Figure 5:**
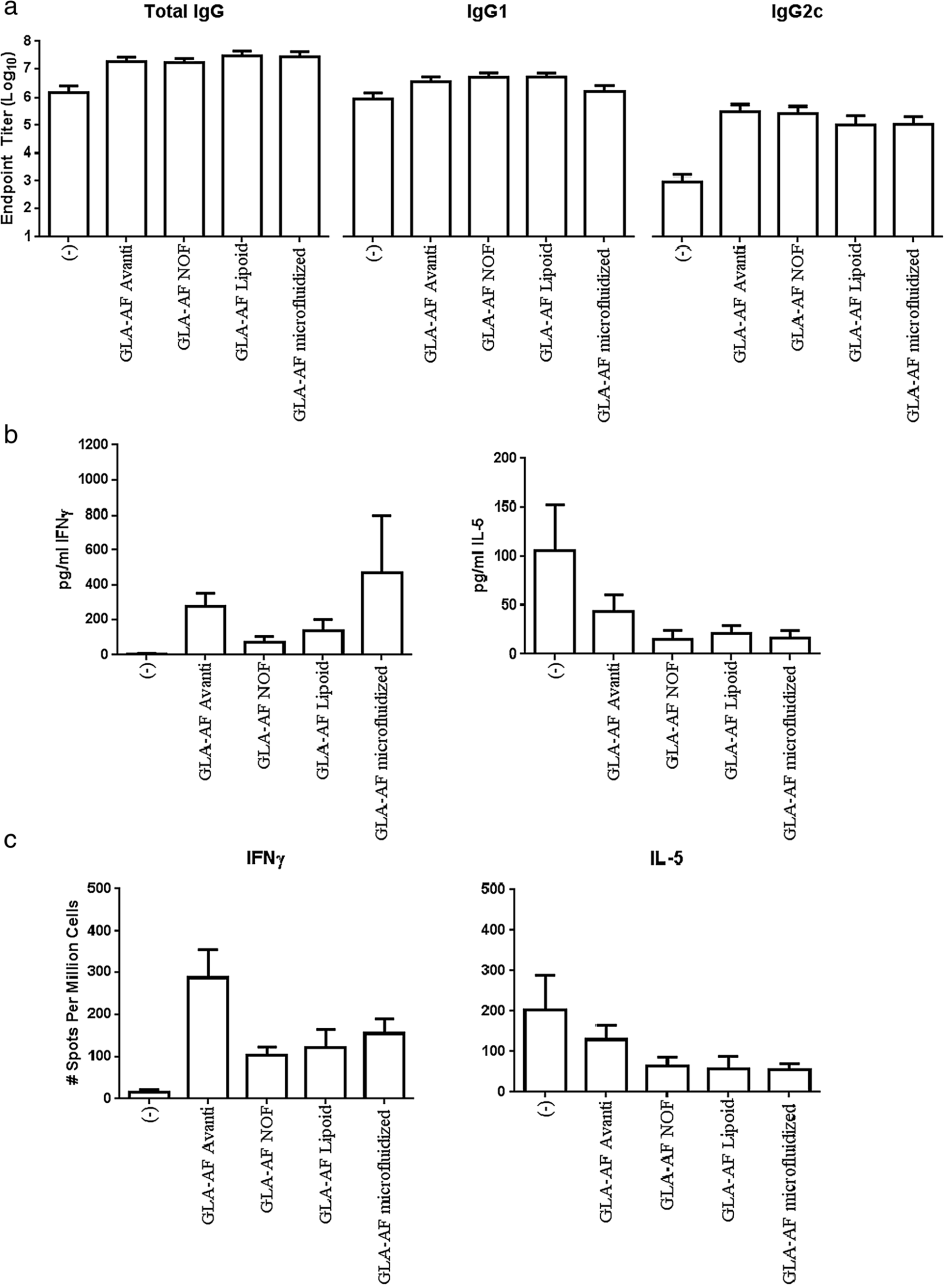
***In vivo *adjuvant activity elicited by PbCSP antigen with GLA-AF. (a)** Antibody endpoint titers measured two weeks after the second immunization. Each group consisted of 4–5 mice immunized with 1 μg GLA-AF and 2 μg PbCSP. Each of the adjuvanted groups achieved statistical significance in IgG2c titers compared to PbCSP alone (p < 0.05). No other statistical differences between groups were apparent. **(b)** Cytokine ELISAs of splenocytes collected six weeks after the third immunization and stimulated with antigen. Each group consisted of 5 mice immunized with 1 μg GLA-AF and 2 μg PbCSP, with each value consisting of an average of two duplicates. No statistical differences between groups were present. **(c)** Splenocytes producing cytokines six weeks after the third immunization, detected by ELISPOT assay. Each group consisted of 5 mice immunized with 1 μg GLA-AF and 2 μg PbCSP, with each value consisting of an average of two duplicates. The number of IFNγ-producing cells from the PbCSP + GLA-AF Avanti group acheived statistical significance (p < 0.05) compared to PbCSP alone, PbCSP + GLA-AF NOF, and PbCSP + GLA-AF Lipoid. No other statistical differences between groups were apparent.

To determine how GLA-AF formulation parameters influenced qualitative antigen-specific immune responses, we immunized mice (n = 4-5) with recombinant protein (PbCSP) mixed into various GLA-AF formulations. As expected, PbCSP immunization raised antigen-specific IgG and administration with adjuvant elevated these responses only marginally. Upon further analyses, it was evident that, while antigen-specifc IgG1 responses were unaltered, inclusion of any of the various GLA-AF significantly increased IgG2c antibody titers compared to administration of the antigen alone (Figure 5a). These data are indicative of the Th1-biasing activity of GLA-containing formulations. This observation was corroborated by analyses of the cellular responses. Antigen-specific recall responses demonstrated a trend of IL-5 secretion reduction and IFN-γ secretion enhancement in mice immunized with GLA-AF formulations relative to mice treated with protein alone (Figure 5b). Similar observations were made when the number of IL-5 and IFN-γ secreting cells were determined (Figure 5c). Taken together, the pattern of antibody and cellular responses indicate that each of the GLA-AF evaluated is capable of biasing toward antigen-specific Th1 responses. Thus, excipient source and manufacturing method did not appear to alter the qualitative immunogenicity response induced by the adjuvanted vaccine. These data, generated with a 1 μg dose GLA, are consistent with our previous reports generated using higher (5–20 μg) doses [9].

In summary, the source of DPPC appears to have minimal impact on the physicochemical characteristics and biological activity of GLA-AF. Buffered compositions may have slightly decreased the tendency for pH drift over time, but overall these resulted in decreased particle size stability compared to unbuffered GLA-AF. Microfluidization resulted in rapid particle size reduction after only a few passes, and 20,000 or 30,000 psi processing pressures were more effective at reducing particle size than 10,000 psi. Microfluidized batches maintained good particle size and chemical (GLA concentration) stability over 6 months, without significantly altering *in vitro* bioactivity of GLA-AF or *in vivo* bioactivity of GLA-AF when combined with a recombinant vaccine antigen. However, some microfluidization batches resulted in lower recovery of GLA depending on process pressure. We note here that concentrations of GLA > 0.04 mg/ml are unlikely to be necessary in large scale batches given the low clinical doses employed (≤10 μg). GLA-AF is currently being evaluated as a vaccine adjuvant formulation in various phase I clinical trials. Microfluidization may be an effective manufacturing process to improve the scalability and reproducibility of GLA-AF as it advances further in the clinical development pathway. Process optimization work including temperature control is ongoing in our lab and may be applicable to other nanosuspension-based formulations.

## Competing interests

The authors declare that they have no competing interests.

## Authors’ contributions

HWMF carried out sonication manufacturing, particle size and pH measurements, HPLC-CAD, and drafted the manuscript. TJTM carried out microfluidization manufacturing, particle size measurement, HPLC-CAD, and drafted the manuscript. JV carried out the *in vivo* immunizations and measured the resulting antibody and cellular assays. SJS and JG designed and carried out the *in vitro* bioactivity assays. MSD designed the *in vivo* immunogenicity study and drafted the manuscript. TSV contributed experimental design and edited the manuscript. CBF designed experiments and drafted the manuscript. All authors read and approved the final manuscript.

## Supplementary Material

Additional file 1: Figure S1 Differential scanning calorimetry scans of microfluidized or sonicated GLA-AF. Click here for file

## References

[B1] Fox CB Friede M Reed SG Ireton GC Wang X, Quinn PJ Synthetic and natural TLR4 agonists as safe and effective vaccine adjuvants Endotoxins: Structure, Function and Recognition, Volume 53 2010 New York: Springer 303 321 Harris JR, Quinn PJ (Series Editor): *Subcellular Biochemistry* 10.1007/978-90-481-9078-2_14 20593273

[B2] Orr MT Fox CB Baldwin SL Sivananthan SJ Lucas E Lin S Phan T Moon JJ Vedvick TS Reed SG Coler RN Adjuvant formulation structure and composition is critical for the development of an effective vaccine against tuberculosis J Control Rel 2013 11 190 200 10.1016/j.jconrel.2013.07.030 PMC3871206 23933525

[B3] Fox CB Kramer RM Barnes VL Dowling QM Vedvick TS Working together: interactions between vaccine antigens and adjuvants Ther Adv Vaccines 2013 11 7 20 10.1177/2051013613480144 PMC3967670 24757512

[B4] Fox CB Characterization of TLR4 agonist effects on Alhydrogel sedimentation: a novel application of laser scattering optical profiling J Pharm Sci 2012 11 4357 4364 10.1002/jps.23307 22927211

[B5] Fox CB Baldwin SL Vedvick TS Angov E Reed SG Effects on immunogenicity by formulations of emulsion-based adjuvants for malaria vaccines Clin Vaccine Immunol 2012 11 1633 1640 10.1128/CVI.00235-12 22896687 PMC3485880

[B6] Fox CB Haensler J An update on safety and immunogenicity of vaccines containing emulsion-based adjuvants Expert Rev Vaccines 2013 11 747 758 10.1586/14760584.2013.811188 23885820

[B7] Reed SG Bertholet S Coler RN Friede M New horizons in adjuvants for vaccine development Trends Immunol 2009 11 23 32 10.1016/j.it.2008.09.006 19059004

[B8] Fox CB Moutaftsi M Vedvick TS Coler RN Reed SG TLR4 ligand formulation causes distinct effects on antigen-specific cell-mediated and humoral immune responses Vaccine 2013 in press, doi:10.1016/j.vaccine.2013.09.069 10.1016/j.vaccine.2013.09.069 24120675

[B9] Misquith A Fung M Dowling QM Guderian JA Vedvick TS Fox CB *In vitro* evaluation of TLR4 agonist activity: formulation effects Colloids Surf B Biointerfaces 2013 11 312 319 24121074 10.1016/j.colsurfb.2013.09.006 PMC3877169

[B10] Sipai ABM Vandana Y Mamatha Y Prasanth VV Liposomes: an overview J Pharm Sci Innov 2012 11 13 21

[B11] Wagner A Vorauer-Uhl K Liposome technology for industrial purposes J Drug Del 2011 11 591325 10.1155/2011/591325 PMC3065896 21490754

[B12] O'Hagan DT Ott GS Van Nest G Rappuoli R Giudice GD The history of MF59 adjuvant: a phoenix that arose from the ashes Exp Rev Vaccines 2013 11 13 30 10.1586/erv.12.140 23256736

[B13] Vandepape-Liere P Novel composition WIPO 2007/068907 A2, GlaxoSmithKline, 21 June 2007

[B14] Kim SH Park Y Matalon S Franses EI Effect of buffer composition and preparation protocol on the dispersion stability and interfacial behavior of aqueous DPPC dispersions Colloids Surf B Biointerfaces 2008 11 253 260 10.1016/j.colsurfb.2008.09.003 18930639

