# Supplementary material for: Optimizing manufacturing and composition of a TLR4 nanosuspension: physicochemical stability and vaccine adjuvant activity
Source: J Nanobiotechnology. 2013 Dec 21;11:43. doi: 10.1186/1477-3155-11-43 (PMC3881025; doi:10.1186/1477-3155-11-43)
Supplement: Additional file 1: Figure S1 — Differential scanning calorimetry scans of microfluidized or sonicated GLA-AF. [file 1477-3155-11-43-S1.pptx]

## Slide 1
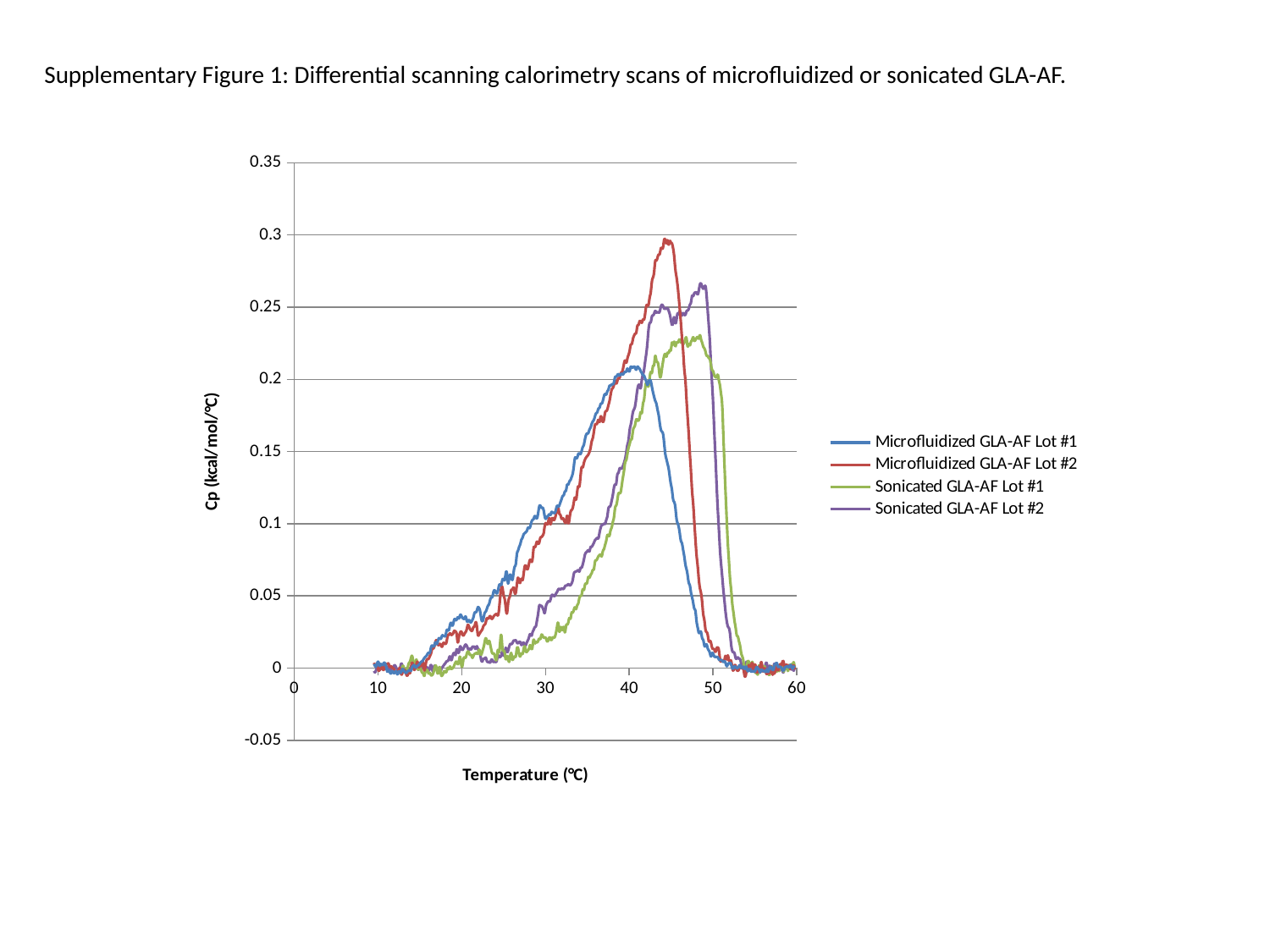

Supplementary Figure 1: Differential scanning calorimetry scans of microfluidized or sonicated GLA-AF.
### Chart
| Category | Microfluidized GLA-AF Lot #1 | Microfluidized GLA-AF Lot #2 | Sonicated GLA-AF Lot #1 | Sonicated GLA-AF Lot #2 |
|---|---|---|---|---|
